# Epithelial vectorial ion transport in cystic fibrosis: Dysfunction, measurement, and pharmacotherapy to target the primary deficit

**DOI:** 10.1177/2050312120933807

**Published:** 2020-06-25

**Authors:** Lucy A Clunes, Naia McMillan-Castanares, Neil Mehta, Afia Mesadieu, Jorge Rodriguez, Mary Maj, Mark T Clunes

**Affiliations:** 1 Department of Pharmacology, St. George’s University, Grenada, West Indies; 2 Medical Student Research Institute, St. George’s University, Grenada, West Indies; 3 Department of Biochemistry, St. George’s University, Grenada, West Indies; 4 Department of Physiology, Neuroscience and Behavioral Sciences, St. George’s University, Grenada, West Indies

**Keywords:** Respiratory medicine, pharmacoeconomics/health economics, ion transport

## Abstract

Cystic fibrosis patients display multi-organ system dysfunction (e.g. pancreas, gastrointestinal tract, and lung) with pathogenesis linked to a failure of Cl^−^ secretion from the epithelial surfaces of these organs. If unmanaged, organ dysfunction starts early and patients experience chronic respiratory infection with reduced lung function and a failure to thrive due to gastrointestinal malabsorption. Early mortality is typically caused by respiratory failure. In the past 40 years of newborn screening and improved disease management have driven the median survival up from the mid-teens to 43–53, with most of that improvement coming from earlier and more aggressive management of the symptoms. In the last decade, promising pharmacotherapies have been developed for the correction of the underlying epithelial dysfunction, namely, Cl^−^ secretion. A new generation of systemic drugs target the mutated Cl^−^ channels in cystic fibrosis patients and allow trafficking of the immature mutated protein to the cell membrane (correctors), restore function to the channel once in situ (potentiators), or increase protein levels in the cells (amplifiers). Restoration of channel function prior to symptom development has the potential to significantly change the trajectory of disease progression and their evidence suggests that a modest restoration of Cl^−^ secretion may delay disease progression by decades. In this article, we review epithelial vectorial ion and fluid transport, its quantification and measurement as a marker for cystic fibrosis ion transport dysfunction, and highlight some of the recent therapies targeted at the dysfunctional ion transport of cystic fibrosis.

## Method

This is a topical review of the physiology of ion transport in cystic fibrosis (CF) and a systematic review of the ion transport pharmacotherapies that have completed phase III clinical trials and aims to summarize the efficacy outcomes of pulmonary function tests (forced expiratory volume in 1 s (FEV_1_)) and ion transport activity (sweat chloride). The drug candidates selected were identified from those in the North American CF drug development pipeline that had completed phase III clinical trials and that are specifically targeted at CF transmembrane conductance regulator (CFTR) ion transport restoration through channel placement or channel modification. The four leading drug candidates from the CF drug development pipeline were searched in Pubmed (between December 2018 and May 2020) (ivacaftor OR tezacaftor OR lumacaftor OR orkambi OR symdeko OR elexacaftor OR symdeko AND clinical trial, returning 118 results) Duplicates were removed and abstracts screened for efficacy outcome FEV_1_. Thirty-five reports remained. Papers with safety data only were excluded. After reviewing the full texts, the papers retained (23) included those with one or more of the measurable patient outcomes FEV_1_ and also for other measures such as ion transport (sweat test results), exacerbation rates, or change in patient’s body mass index (BMI). The 23 papers that include these data are presented in Table 1. The major limitations of this approach are that the drug candidates are delineated by the CF drug development pipeline and have been through phase III clinical trials. The data are not always comparable between studies because of the variant time and dose regimens, but the endpoint efficacy data are included for comparison. In addition, the genotypes are often not fully known for heterozygous CF patients but the inclusion criterion is reported for each study. For FEV_1_ data, the absolute changes are recorded along with placebo changes, and where data are only relative to placebo, it is indicated in the table by placebo effect of 0%. Therefore, this review is a summary of the available data on patient’s pulmonary function changes (delta FEV_1_) and its association with ion transport correction (sweat chloride concentration changes) and quality of life (BMI and exacerbations) for CF patients enrolled in phase III trials of the major ion transport monotherapies and combinatorial therapies for restoration of ion transport through CFTR channels.

**Table 1.** Summary of pulmonary function endpoint changes in FEV_1_ and ion transport effects (sweat chloride) as well as indexes of patients’ quality of life (BMI and exacerbations) during corrector and potentiator therapy in CF patients.

Drug	Duration	Δ FEV_1_ (%)	Δ FEV_1_ placebo/cont. (%)	Δ sweat Cl^−^ (%)	Δ BMI	Exacerbations	References
ΔF508 heterozygous							
Elexacaftor–tezacaftor–ivacaftor	24 weeks	13.90	−0.20	−42.00	1.13 (5.3%)	0.37	Middleton et al.^1^
VX445–tezacaftor–ivacaftor	29 days	11.00	0.40	−40.00	–	–	Keating et al.^2^
VX-659–tezacaftor–ivacaftor	4 weeks	13.30	0.40	−51.00	–	–	Davies et al.^3^
Tezacaftor–ivacaftor	8 weeks	6.80	0	−14.80	–	0.29	Rowe et al.^4^
Ivacaftor	8 weeks	4.70	0	−6.00	–	0.34	Rowe et al.^4^
Lumacaftor–ivacaftor	56 days	−0.60	−1.20	–	-0.04	–	Rowe et al.^5^
Lumacaftor (monotherapy) to lumacaftor–ivacaftor	56 days	−1.70	−2.00	−5.20	–	–	Boyle et al.^6^
ΔF508 homozygous							
Elexacaftor added to tezacaftor–ivacaftor	4 weeks	10.40	0.4% control	−47.50	–	–	Heijerman et al.^7^
VX445–tezacaftor–ivacaftor	29 days	13.80	0	−40.00	–	–	Keating et al.^2^
VX-659–tezacaftor–ivacaftor	4 weeks	9.70	0	−42.00	–	–	Davies et al.^3^
Tezacaftor–ivacaftor	24 weeks	3.40	−0.60	−10.00	0.18 (0.8%)	0.64	Taylor-Cousar et al.^8^
Tezacaftor	28 days	3.49	−0.14	−20.40	–	–	Donaldson et al.^9^
Tezacaftor–ivacaftor	28 days	3.75	−0.14	−6.00	–	–	Donaldson et al.^9^
Lumacaftor–ivacaftor	24 weeks	4.00	0	–	0.4 (1.9%)	0.61	Wainwright et al.^10^
Lumacaftor–ivacaftor	24 weeks	1.10	−1.30	−20.00	0.4 (2.4%)	–	Ratjen et al.^11^
Lumacaftor–ivacaftor	24 weeks	2.50	–	−24.80	0.64 (3.8%)	–	Milla et al.^12^
Lumacaftor–ivacaftor	24 weeks	2.20	−0.40	–	0.37	0.61	Konstan et al.^13^
Lumacaftor–ivacaftor maintenance	96 weeks	0.80	–	–	0.96	0.61	Konstan et al.^13^
Lumacaftor–ivacaftor	24 weeks	3.70	0	–	0.6	–	Elborn et al.^14^
Lumacaftor (monotherapy) to lumacaftor–ivacaftor	56 days	3.60	−2.0	−8.90	–	–	Boyle et al.^6^
Lumacaftor–ivacaftor	11 months	0.10	–	–	–	–	Jennings et al.^15^
Lumacaftor–ivacaftor	8–16 weeks	2.30	–	−20	–	–	Graeber et al.^16^
Ivacaftor	16 weeks	1.50	−0.20	−75	–	–	Flume et al.^17^
ΔF508/G551D							
Tezacaftor	28 days	1.40	-0.14	−10.20	–	–	Donaldson et al.^9^
Tezacaftor–ivacaftor	28 days	4.60	−0.14	−7.00	–	–	Donaldson et al.^9^
Ivacaftor	48 weeks	10.60	−0.20	−50.00	1.08 (4.9%)	–	Ramsey et al.^18^
Ivacaftor	48 weeks	12.50	0.10	−48.00	2.03 (11%)	–	Davies et al.^19^
G551D heterozygous							
Ivacaftor	28 days	7.00	0	−45	–	–	Davies et al.^20^
Ivacaftor	24 weeks	5.50	–	–	5%	–	Taylor-Cousar et al.^21^
Ivacaftor	48 weeks	9.40	−1.20	–	5%	0.46	McKone et al.^22^
Ivacaftor	96 weeks	9.50	–	–	*–*	–	McKone et al.^22^
Ivacaftor	144 weeks	9.40	–	–	*–*	–	McKone et al.^22^
G551D/R117H							
Ivacaftor	24 weeks	2.60	0.50	−26.30	–	–	Moss et al.^23^
Non-G551D/DF508							
Ivacaftor	8 weeks	2.0/19.0^a^	−3.20	−6/−80^a^	0.16/1.62^a^	–	De Boeck et al.^24^

BMI: body mass index; CF: cystic fibrosis; FEV_1_: forced expiratory volume in 1 s.

a By genotype.

## Introduction and background

More than 30,000 Americans live with CF, the most common autosomal recessive genetic disorder of Caucasians. In North European derived populations, the carrier frequency is 1/27 and live birth prevalence is 1/2500–1/4000.^25^ The symptoms most commonly reported at diagnosis are acute or persistent respiratory abnormalities (36%), failure to thrive (28%), steatorrhea/abnormal stool/malabsorption (21%), and meconium ileus/intestinal obstruction (17%).^26^ Most patients experience respiratory failure in early adulthood after years of chronic respiratory infection and inflammation caused by persistent bacterial colonization of the airway lumen. In 1970, the median age of survival for a person with CF was around 10 years, since that time, there has been an improvement in diagnostic screening, an increase in the array of available therapies and improved patient management, nutrition, and education and a concomitant increase in the median survival to 43–53 years.^26,27^

CF disease of ΔF508 patients is characterized by reduced pancreatic function, associated with malabsorption, a failure to thrive, and stool abnormalities such as steatorrhea within the first 6 months of life, and the majority of patients show pancreatic insufficiency by 12 months of age.^28^ This is associated with sinopulmonary manifestations throughout early childhood; recurrent respiratory infections, chronic cough, wheeze, and pneumonia/sinusitis; and the later development of bronchiectasis. Lung disease is initially characterized as obstructive and with time may show elements of both obstructive and restrictive diseases, especially in the latter stages. The development of gallstones, liver disease, and diabetes may also occur in later disease. Overall, the rate of pulmonary disease progression is variable though inevitable. There is a poor correlation between genotype and respiratory disease progression and severity, and recognition that a late diagnosis based on symptoms is associated with worse patient outcomes overall. This has driven an increase in newborn screening to provide early diagnosis of CF and initiation of therapy prior to CF symptom onset.

A gene (NM 000492) 30 years ago was associated with the syndrome of CF and within 2 years of its identification, it was cloned and its product was identified as a plasma membrane bound cyclic-adenosine monophosphate (cAMP) sensitive anion channel; the expression of which could correct ion transport in CF airway cells,^29^ as a result, it was called the CFTR. Absence or dysfunction of this protein or its regulation leads to the symptomology of CF, and to date, >2000 mutations in the CFTR gene have been documented.^30^

**Figure 1.**
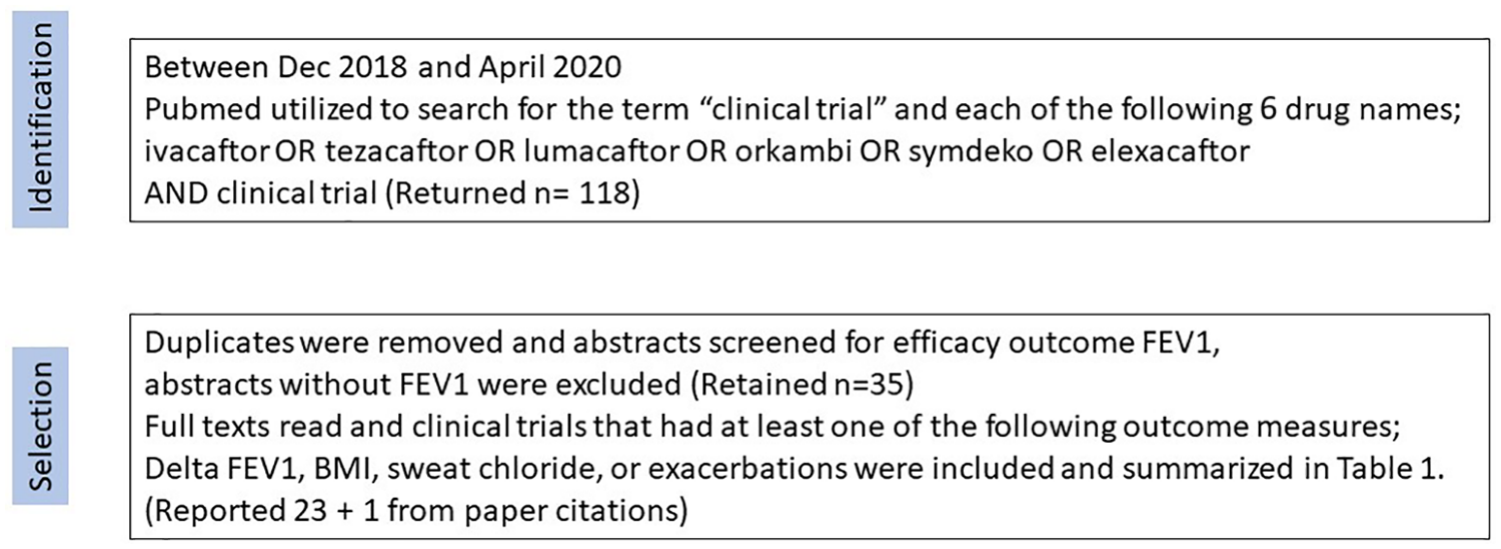
(a) The majority of secretory epithelia show chloride mediated ion secretion. The Na-K-ATPase generates an inwardly directed Na^+^ gradient, and along with the negative resting membrane potential augments the driving force for Na^+^ entry to the cell. The basolateral triple co-transporter uses sodium’s large driving force to co-transport one K^+^ ion and two Cl^−^ ions. Cl^−^ is accumulated inside the cell above its electrochemical equilibrium and the apical CFTR channel allows the efflux of Cl^−^ ions into the lumen, creating a lumen negative trans-epithelial potential difference. The negative potential in the lumen provides an electrical driving force for Na^+^ to flow paracellularly, and with the accumulation of NaCl in the lumen, the osmotic gradient for water flux is established. The apical Cl^−^ conductance can be CFTR or other regulated Cl^−^ conductances, for example, calcium-activated chloride conductance. In order for sustained Cl^−^ efflux to the lumen the cell interior must be hyperpolarized beyond the Cl^−^ equilibrium potential; opening K^+^ channels allows this hyperpolarization and drives Cl^−^ ions into the lumen. In some epithelia, for example, airway, ENaC channels in the apical membrane allow the epithelium to sustain absorption when required. This demands coordination of the two apical conductances; when secretion is taking place (as described above), it is crucial that the apical ENaC channel is quiescent, and the CFTR channel is known to play a role in this regulation through direct protein–protein interaction. When the CFTR channel is quiescent and the ENaC channel is activated, this allows Na^+^ to flow out of the lumen into the cell, down its electrochemical gradient, establishing a negative potential in the lumen. The intracellular Na^+^ is pumped to the basolateral space by the Na/K-ATPase. This negative lumen potential drives Cl^−^ ions paracellularly and achieves a bulk transport of salt from the lumen to the basolateral space and an osmotic gradient for water flux. (b) In some specialized epithelia, like the reabsorptive duct of the sweat gland, ENaC and CFTR demonstrate co-transport, see main text for details.

To understand the likely pathogenic mechanisms in CF, it is necessary to understand how the airway epithelium regulates ion and fluid secretion under normal circumstances and the role of the CFTR channel in that activity. A schematic representation of the airway epithelium is shown in Figure 1(a). The ciliated surface epithelium of the respiratory tract is the main surface area in contact with the airway surface liquid. Fluid may be secreted by the alveolar epithelium and by the submucosal glands^31^ but the surface epithelium with its high contact surface area is the best place to modify and regulate the fluid composition and depth of the airway surface liquid. The airway surface epithelial cells express the essential channels required for vectorial transport of salt across the epithelium^32,33^ and have both secretory and absorptive capacities. Not all epithelia are equivalent, for example, in the GI tract, there is regionalization of CFTR and epithelial Na^+^ channel (ENaC), so that there is either secretory or absorptive capacity in any one geographical region of epithelium. The surface airway epithelium, however, can switch between secretion and absorption and demonstrates a complex interplay between CFTR and ENaC to achieve coordinated channel activity and a net vectorial transport of salt with temporal differentiation of secretion or absorption in the same piece of epithelium.

The CFTR gene was presciently named, its protein product is an anion channel regulated by cAMP/protein kinase A (PKA), and its gating is clearly linked to the control of an anion conductance (primarily Cl^−^) involved in salt and fluid secretion. However, subsequent experiments also established that the CFTR protein can regulate other conductances in cells, one of which is ENaC.^34^ Not all epithelia express CFTR and ENaC in the same cells, so the regulation of ENaC by CFTR is epithelium-dependent and requires co-expression. In addition, the role of the epithelium also determines the type of regulation that takes place between these two channels, for example, the co-regulation of CFTR and ENaC is different when comparing the reabsorptive duct of the sweat glands and the surface airway epithelium, both of these epithelia co-expresses CFTR and ENaC and yet the co-regulation of the two channels is quite different in these two settings:^35^ in the sweat gland reabsorptive duct, both channels actively work together to mediate absorption (see Figure 1(b)), while in the surface airway epithelium, each channel has a predominant role in sustaining absorption (ENaC) or secretion (CFTR). The unique role of CFTR in the reabsorptive duct of the sweat gland will be examined in a later section.

In the pulmonary epithelium, around which much of the symptomology of CF surrounds, there is a critical link between CFTR and ENaC function that makes the absence of CFTR more insidious than a mere lack of secretion. In the pulmonary epithelium, in particular, the ciliated airway surface cells, CFTR may play a modulatory role for ENaC function by balancing secretory and absorptive processes across the airway surface. CFTR when active provides an inhibitory influence upon ENaC activity and allows an integrated response of the epithelium for vectorial ion transport, so that Cl^−^ secretion and Na^+^ absorption do not occur together. When CFTR is active, Cl^−^ is transported out of the cells into the airway lumen and generates a lumen negative trans-epithelial potential that drives Na^+^ paracellularly through the “leaky” airway epithelium. In this way, a mass of salt is vectorially transported from the basolateral to the luminal space and provides the osmotic driving force for water transport, thus sustaining fluid secretion. CFTR’s negative feedback upon ENaC, which may be mediated by a direct protein–protein interaction, keeps the ENaC channels “quiet” during this secretory response and suppresses absorptive activity. However, when CFTR is “switched off” and ENaC is “switched on” the epithelium shifts from net secretion to net absorption. When CFTR is “quiet,” Na^+^ is absorbed from the lumen into the cells through ENaC and pumped into the basolateral space by the Na-K-ATPase, thus forming a lumen negative trans-epithelial potential that drives Cl^−^ paracellularly from the lumen to the basolateral space. This switches the epithelium to a salt absorbing mode and the movement of salt from the luminal to basolateral space forms the osmotic driving force for water movement out of the lumen and into the basolateral domain (see Figure 1(a)).

Thus, an epithelium that expresses both CFTR and ENaC can switch between secretion and absorption by temporally separating out the activity of the CFTR and ENaC channels, and this regulation may be coordinated through direct protein–protein interaction (or intermediary protein) between CFTR and ENaC. In CF patients with homozygous ΔF508 mutation, the CFTR gene product never makes it to the plasma membrane. The absence of CFTR from the membrane removes the inhibitory action of CFTR on ENaC activity and allows ENaC to enter a hyperactive, hyper-absorptive state. Therefore, in airways, it is not only the lack of secretion that drives CF airway pathogenesis but also the enhanced salt absorption through ENaC leads to extreme mucus inspissation with enhanced adherence to the airways. This is borne out experimentally where over-expression of ENaC alone shifts epithelial function toward net absorption and results in thick adherent mucus and airway inflammation.^36^ The viscid adherent mucus suppresses normal mucociliary clearance mechanisms by inhibiting ciliary action. This leads to mucus stasis, and since mucus is a sugar-coated protein polymer gel, it provides a stable substrate for bacterial growth and persistent biofilm development, with subsequent inflammation and remodeling of airways. The essential property of mucus as a barrier protecting the airways is that the polymer gel is loose, hydrated, and mobile; the mucus mesh then entraps pathogens and mucociliary activity moves the mucus/pathogen load up and out of the airways for voidance. The absence of CFTR-mediated Cl^−^ and fluid secretion occurs across many epithelia in CF patients. CF is a multi-organ/tissue disorder, and respiratory failure has been the leading cause of mortality, to date, but as median survival has increased, there is an increased requirement to manage other comorbidities. Most of the other pathogenic processes are directly related to the lack of secretion in CF or the inflammatory damage done in tissues in response to static inspissated mucus, and the morbidities prevalence generally get worse with age.

CF-related diabetes (CFRD) has been reported in 2% of the CF population at age <10 years, at around 11% at age 20, and rises to 35%–50% in patients older than 30–35 years.^37,38^ Likewise, CF-related liver disease (CFLD) is generally age dependent, although if present it typically has earlier onset and a more rapid progression than CFRD.^39^ Gastroesophageal reflux disease (GERD), whether symptomatic or “silent,” is also more common in CF patients and may be present in as much as 90% of the CF population.^40^ In addition to the pathogenic disease mechanisms, the long-term use of therapies such as antibiotics and inadequacy of pancreatic enzyme supplementation also serve to exacerbate gastrointestinal (GI) complications. Distal intestinal obstruction syndrome (DIOS) is also more prevalent in CF patients and primarily occurs in adolescents and young adults.^41^ Constipation, rectal prolapse, intussusception, small intestinal bacterial overgrowth, intestinal inflammation, cholelithiasis, cholecystitis, hepatic steatosis, pancreatic insufficiency, and pancreatitis are relatively common GI complications that need to be managed.

Until recently, the main pharmacotherapies for CF were focused on symptom management, for example, mucolytics (DNAse and osmotic agents such as hypertonic saline and mannitol), bronchodilators for airway management, enzyme supplements, and nutritional management for GI issues. Although the promise of gene therapy has been explored since the late 1980s, there remains to be any major breakthrough that has advanced to clinical use, and experimental studies report modest and transient functional correction. However, the most promising development in the last 10–15 years has been in systemic pharmacotherapies aimed at the correction of the primary ion transport defect in Class II mutations of CFTR or in normalizing the gating abnormalities present in mutated CFTR that is resident in the membrane. As early as the mid-1990s, reports of experimental studies in vitro causing the augmentation of CFTR’s function in response to genistein were reported^42^ and later reports in the early 2000s suggested curcumin was also able to augment Cl^−^ channel function.^43^ These reports were the first suggestion that pharmacotherapeutically CFTR itself could be the target, for trafficking to the membrane and augmentation of function. This started a major screening initiative that aimed to identify agents capable of stabilizing and augmenting CFTR function in vivo. In the case of the most common mutation ΔF508, a combined therapy would be required to restore function, as chaperoning of the protein to the membrane as well as increasing channel function would be required for functional restoration of the channel activity. This has been the goal of concerted corrector and potentiator pharmacotherapy over the last 10–15 years. The most rapid progress has been made with potentiators; drugs that can restore some function to mutated protein that is already resident in the plasma membrane. The mutations G1244E, G134GD, G178R, G551D, G551S, R117H, S1251N, S549N, and S549R are all Class III or IV mutations and all have reduced open probability (problems with channel opening and closing) or reduced single channel conductance (problems allowing Cl^−^ to flow through the channel). All of these mutations can be targeted for potentiation of function. To date, all have been reported as having potentiated ion transport function and improved patient outcomes with Ivacaftor (Trade name: Kalydeco).^44–47^

To develop drugs for correction or potentiation of CFTR function, it was crucial that more proximal measures of ion transport restoration were available for assessing drug efficacy. In CF, although it is crucial that any drug in development is able to modulate and control symptom severity or retard development of the symptoms; with a chronic progressive disease such as CF, early drug candidates must be tested using more immediate markers of putative drug potential to allow rapid identification of those drugs with most promise. It is fortunate that in CF there are two relatively easy and immediate measures of Cl^−^ ion transport available to determine if CFTR function is augmented such as the sweat Cl^−^ test and the nasal potential difference test. Although neither test indicates that the drugs will change the respiratory function or any other associated morbidity in patients; both of these tests provide an immediate measure of ion transport activity through CFTR and allow assessment of CFTR function in situ in human nasal epithelial cells or in the cells of the reabsorptive duct of the sweat gland. The assumption is that if there is pharmacotherapeutic correction of ion transport in these instances, then all equivalently regulated secretory epithelia will be equally benefited in the patient. However, it may take longer to see the effects on respiratory or GI function, as the primary ion transport defect in these organs starts a cascade of pathophysiology that ultimately culminates in secondary complications around organ inflammatory response and remodeling. In this case, correction of the primary ion transport defect may not re-establish lost function, but in the best cases, it may prevent further decline of function. The long-term goal of such therapies then is to start intervention prior to the development of symptoms that indicate organ dysfunction and focus on delaying tissue damage and symptom development in the first place.

### Sweat Cl^−^ tests

Sweat Cl^−^ tests have been used diagnostically for CF over decades,^30,48^ and the results with ΔF508 mutations are relatively unambiguous. However, more recently, the later diagnosis of CF due to compound heterozygotes and milder loss of function mutations, like G551D and R117H have required reconsideration of the sweat Cl^−^ concentrations used for CF diagnosis. The median sweat Cl^−^ content is 100 mmol/L (~92 mmol/L for the 75th percentile) for ΔF508 homozygous CF patients, but non-homozygous ΔF508 individuals have a lower sweat Cl^−^ content of ~89 mmol/L (~60 mmol/L for the 75th percentile). Sweat Cl^−^ concentration for ΔF508 individuals has remained stable since 1997 reflecting a stable well identified patient population, however, non-homozygous ΔF508 individuals have seen a drift to lower sweat Cl^−^ test values in that same period, indicating an increased frequency of milder loss of function mutations and compound heterozygotes in the registry.^26^ Nevertheless, the sweat test, along with nasal potential difference measurement, represents functional tests that can measure ion transport and its deficiency in CF patients.

### Rationale for the sweat test

In humans, eccrine sweat glands are primarily thermoregulatory and sympathetic cholinergic mediated, secreting a very hypotonic fluid onto the skin surface with low sweat rates. The sweat glands are composed of two segments such as the secretory coil within the dermis and the reabsorptive duct that traverses the epidermis and opens onto the skin surface. The secretory coil of the sweat gland secretes an isotonic fluid into the gland lumen, and myoepithelial cell contraction helps to drive the tubular fluid through the secretory coil in the dermis and up through the reabsorptive duct in the epidermis. The Cl^−^ channels that regulate fluid secretion in the secretory coil are primarily calcium-activated Cl^−^ channels, and it is likely that CFTR plays only a subsidiary role, if any, in the secretion of sweat from the secretory coil. As the isotonic fluid from the secretory coil travels through the reabsorptive duct, an electrically tight epithelium, CFTR and ENaC channels play an important role in the absorption of Na^+^ and Cl^−^ from the luminal isotonic solution (Figure 1(b)). The tight epithelium prevents any paracellular flow of ions or water and limits trans-epithelial reabsorption of water despite active salt absorption, resulting in a luminal fluid that becomes hypotonic by the time it reaches the skin surface (<20 mM).^49^ Faster flow rates of sweat through the reabsorptive duct decrease the resident time of the luminal fluid in the duct and give less time for reabsorption; therefore, as sweat rates increase the salt concentration of sweat reaching the skin surface increases toward isotonicity. In the reabsorptive duct of CF patients, the absence of CFTR severely limits Cl^−^ and hence Na^+^ reabsorption from the luminal fluid across the reabsorptive duct, so the fluid making it to the skin surface has a highly elevated Cl^−^ concentration. However, CF patients can secrete sweat normally from the secretory coil of the sweat gland where CFTR function is not critical to sustain Cl^−^-mediated ion secretion. Indeed, M2 receptor-mediated sympathetic cholinergic secretion appears normal in CF patients; however, there is evidence to suggest that beta-adrenergic mediated secretion is deficient in CF patients.^50^ Functionally, this has little effect on sweat secretion in CF patients, since the primary regulator of sweat secretion is sympathetic cholinergic rather than adrenergic. With high sweat rates, CF patients are prone to electrolyte losses, since the sweat reaching the skin surface is closer to isotonic rather than hypotonic. Indeed, the high degree of heat prostration seen in patients with fibrocystic disease of the pancreas during the summer heatwave of 1948 in New York was one event that lead to the discovery that these patients had elevated sweat Cl^−^ and was perhaps the first step toward the Cl^−^ test as a diagnostic gold standard for CF.^48^

Diagnostically, sweat Cl^−^ above 60 mmol/L is accepted as a positive indicator of CF in the presence of a positive newborn screen, clinical features consistent with CF, or a positive family history. With a positive newborn screen and a sweat Cl^−^ <30 mmol/L, the presence of CF is unlikely. Intermediate sweat test results, 30–59 mmol/L, indicate that further genetic testing is required, these intermediate sweat values can indicate a genotype that correlates with a less severe phenotype; a comprehensive diagnostic guideline is presented by Farrell et al.^30^ Sweat testing of newborns with a positive CF newborn screen is recommended in infants >2 kg and who are at least 36 weeks of corrected gestational age or as soon as possible after 10 days of age and before 4 weeks,^26^ the full range of pediatric sweat Cl^−^ reference ranges from this time to adulthood is presented in Collie et al.^48^ In addition to sweat Cl^−^ testing, both nasal potential difference measurement and intestinal current measurement are two other bioassays for CFTR-mediated Cl^−^ secretion. These methods are only available in specialized circumstances and are not typically available as part of the mainstream diagnostic suite of tests for CF. Nasal potential difference measurement can be done in vivo; however, intestinal current measurement requires a rectal biopsy and the mounting of the tissue in a specialized Ussing chamber for current measurement; this is far from easy to accomplish clinically and requires very specialized laboratory personnel and equipment. Both tests are generally used in the context of research and drug development rather than clinical diagnosis.

### Rationale for nasal potential difference measurement

During active epithelial Cl^−^ secretion (through CFTR), the vectorial secretion of Cl^−^ into the lumen sets up a trans-epithelial potential difference (lumen negative). Na^+^ follows paracellularly through the “leaky” airway epithelium and the net transport of salt provides the osmotic gradient for water flux and hence fluid secretion. The magnitude of this trans-epithelial potential is determined by the rate of charge separation across the epithelium that is driven by Cl^−^ secretion; more Cl^−^ secretion results in a more negative trans-epithelial potential and reflects a greater secretory activity. However, ion and fluid absorption results in a similar change in trans-epithelial potential because the secretion of a negative charge into the lumen is electrically equivalent to the absorption of a positive charge out of the lumen. When CFTR is switched off and the ENaC is switched on, the predominant ion transport is absorption of Na^+^ from the lumen to the basolateral space. Na^+^ flows down its electrochemical gradient into the cell down a large electrochemical gradient and is then transported into the basolateral space by the Na-K ATPase. The absorption of positive Na^+^ ions from the luminal to the basolateral space also leaves the luminal space negative and increased rates of Na^+^, and fluid absorption, result in more negative trans-epithelial potentials. In order to discriminate whether a negative potential is due to increased Cl^−^ secretion or increased Na^+^ absorption, it is essential to block one of these pathways and examine the residual potential and its regulation. Typically, amiloride, an ENaC channel blocker, is administered first to block the baseline Na^+^ absorption. This results in a depolarization of the trans-epithelial potential (closer to zero). In the presence of amiloride, CFTR channel activators are then added to stimulate secretion, for example, isoproterenol. Often low Cl^−^ conditions are also used to enhance the driving force for Cl^−^ secretion and augment Cl^−^ mediated ion transport. The stimulation of CFTR activity via the beta2-cAMP/PKA pathway causes a hyperpolarization of the trans-epithelial potential. In a CF patient, the addition of amiloride and the depolarization of the trans-epithelial potential is intact, but the addition of a CFTR activator or low Cl^−^ conditions results in no subsequent hyperpolarization, reflective of the absence of a Cl^−^ current into the luminal space. One other characteristic of the CF nasal potential difference is that the baseline potential is very augmented compared to normal; this reflects a higher than normal Na^+^ absorption in CF patients.^51^ This high baseline potential is almost entirely amiloride-sensitive and indicates that the amiloride-sensitive Na^+^ absorption in CF patients is highly augmented. This reflects hyper-absorption of Na^+^, and likely reflects higher than normal ENaC activity in CF patients due to the absence of the inhibitory regulation of the ENaC channel by the CFTR protein.^34,36,52^ In summary, the trans-epithelial potential of a CF patient is high under baseline conditions, is almost entirely amiloride-sensitive, and fails to increase in response to cAMP/PKA stimulation in the presence of amiloride. The main use of nasal potential difference measurement is for clinical trial drug development rather than as a diagnostic test for CF.

### CF dysfunction: a plethora of possibilities

The symptomology of CF and the dysfunction of CFTR can occur at various levels, from partially functional protein in the membrane to the absolute absence of protein from the cell membrane. The different mutations can result in different patient outcomes, with high variability in the severity of phenotype.

**Figure 2.**
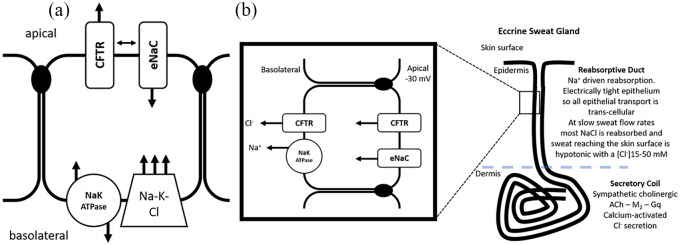
CFTR mutation classes: under normal circumstances, the CFTR gene is transcribed and translated into protein at the endoplasmic reticulum (ER), then processed through the Golgi and moved to the apical membrane; Class I mutants result in no functional CFTR protein generated; Class II mutations allow for the generation of CFTR; however, the protein misfolds which inhibits it from moving to the apical membrane; Class III mutations typically lead to the correct protein structure at the apical membrane; however, the mutations disrupt the channel gate from opening; Class IV mutations allow for the properly folded channel to move to the apical membrane, though show reduced conductance of ions; Class V mutations change the amount of CFTR protein at the apical membrane, due to either partially aberrant splicing or missense mutations that affect trafficking of the protein to the cell surface; Class VI mutations lead to decreased protein stability at the apical membrane and the protein is sent to the lysosome for degradation. For discussion on how the classification system has been developed further by adding functional pharmacological data, see the main text on theratyping.

Mutations can be classified according to mechanism of dysfunction, see Figure 2. See also https://www.cff.org/Care/Clinician-Resources/Network-News/August-2017/Know-Your-CFTR-Mutations.pdf

With Class I and II mutations, there is an absence of any gene product in the membrane, and the most common genotype in the CF population is the ΔF508 Class II mutation. Of the 2000 cataloged mutations, approximately 40% of them result in single amino acid substitutions, 36% of them result in RNA processing errors (nonsense, missense, and frameshift), 14% are neutral variants, and the remainder are made up of undefined outcomes, rearrangements of CFTR, or promoter problems.^53^ In the north European Caucasian, the most common CF causing mutation is ΔF508, with a reported 70%–76% of patients having this Class II mutation. Class II mutations pose a series of problems for pharmacotherapy. There is an absence of CFTR protein in situ in the plasma membrane due to altered CFTR chaperone interactions and degradation of mutated CFTR by the channel probability in vitro and therefore some cells quality control systems.^54^ However, it has been demonstrated that the mutated protein has a functional conductance and open functionality under experimental conditions,^55^ suggesting that if the protein can be trafficked and placed it may restore partial function—or at least be available for functional modification. If mutated protein can be placed in the plasma membrane in situ, there is an opportunity to then modulate the protein activity and provide functional augmentation of protein function pharmacotherapeutically. The potential of this approach is underscored by patient disease heterogeneity based on different genotypes. Patients with homozygous ΔF508 mutations generally have earlier development and worse expression of symptoms, while loss of function mutations like G551D and R117H can have a very different disease trajectory with very late onset symptoms; so late that CF may not be diagnosed until the third to fifth decade of life; indeed, the class of mutation inherited by a patient has been shown to be associated with symptom onset and disease diagnosis,^56,57^ although gene modifiers significantly alter this association.^58,59^ This highlights that in some patients very little functional protein is required in the membrane to make remarkable differences in disease trajectory and management. The pharmacotherapeutic goal is to deliver mutated protein to the plasma membrane, using a “corrector,” and the last decade has seen a concerted effort to target the ΔF508 mutation for correction with drugs such as Lumacaftor and Tezacaftor, both of which have undergone phase III trials and are available to patients. Experimental studies have shown that ΔF508 CFTR when stable at low temperature has a normal single channel conductance but, that is, the open probability is reduced by ~50% from normal (P_o_ reduced from 0.34 to 0.13).^60^ That mutated CFTR can function and, albeit partially, provides the rationale for developing drugs to stabilize the protein and allow its transfer to the plasma membrane and to then potentiate the probability of its opening.

The next most common mutations are G542X (2%–5%) Class I and G551D (~3%) Class III mutation. The mutation class is an important aspect of CF dysfunction when it comes to rational drug design. Class III mutations are present in the plasma membrane and have gating or conductance problems; therefore, these defective proteins can be targeted for “potentiation” of protein function. The G551D mutation has therefore been targeted extensively for this class of novel pharmacotherapy.

### CF foundation drug discovery pipeline—a snapshot of the leading contenders in the pipeline

In 2008, the CF foundation listed a total of 33 drugs in the development pipeline, of which approximately four targeted CFTR for potentiation of its function or placement in the plasma membrane.^61^ These incipient corrector and potentiator drugs were emerging as putative therapies and most of the initial research centered around the potentiation of G551D CFTR. The G551D mutation was chosen as it is a Class III mutation where the protein is resident in plasma membrane and offered the simplest opportunity for targeting single channel conductance/open probability for channel function augmentation. Ten years later, a few of these early drugs are now Food and Drug Administration (FDA) approved (VX-770—Ivacaftor and VX-809—Lumacaftor) and have shown clinical gains in the G551D CF population. Since that time, there has been an expansion and refinement of this pharmacotherapeutic approach to correct and potentiate CFTR channel activity; of the 15 top drugs currently listed in the CF foundations development pipeline, 13 of them are listed as correctors or potentiators (https://www.cff.org/Trials/Pipeline). That these drugs have demonstrated, early clinical benefit has meant that the focus for drug development in North America has moved firmly to combined systemic corrector/potentiator pharmacotherapy and away from gene therapy, topical application therapies targeting the alternative Cl^−^ pathway or its regulation (i.e. intracellular calcium, inhaled ATP/uridine triphosphate (UTP)), or inhibition of ENaC channel activity (amiloride analogs). While all of these therapies were at the forefront of CF research 10 years ago and may still form promising therapies for CF management, the impetus to drive potentiator/corrector therapy developed because of the early clinical benefits observed in the G551D population and the promise of multi-system correction of the primary ion transport deficit with this approach.

The advantage of systemic corrector/potentiator therapy is the putative gain of function for CFTR across all organ systems; to date, the benefits to the respiratory system are studied but data for other major physiological markers of, for example, GI function, are less well reported.^62^ Inhaled therapies are more limited because they provide gain of function for the respiratory epithelium alone, are difficult to administer uniformly throughout the respiratory tree, and may have limited access to the areas of the respiratory tree where disease pathogenesis is most advanced. Mucus hypersecretion and airway obstruction, as well as the airway remodeling seen in CF, place limitations on access of inhaled drug to the sites that most need the pharmacotherapy. Therefore, systemic drugs aimed at correcting CFTR and potentiating protein function have become the preferred development targets. The correctors currently listed are lumacaftor (available in combination with the potentiator ivacaftor, trade name: Orkambi), tezacaftor (available in combination with the potentiator ivacaftor, trade name: Symdeko), elexacaftor (VX-450; available with tezacaftor and lumacaftor, trade name: Trikafta), VX-659 (phase III), FDL-169 (phase II), GLPG-2222 (phase II), VX-152 (phase II), VX-440 (phase II), VX-445 (phase II), and PTI-801 (phase I). The potentiators are ivacaftor (trade name: Kalydeco), QBW251 (phase II), VX-561 (phase II), and PT-808 (phase I).

A summary of the gains provided by corrector/potentiator drugs that have completed phase III trials is presented in Table 1. Sweat Cl^−^ reduction, exacerbation frequency, and FEV_1_ improvement are the indexes generally studied for the different CF genotypes. In general, potentiator drugs are able to demonstrate a 30%–50% reduction in sweat Cl^−^ for Class III mutations like G551D within days of administration, indicating a rapid and immediate resolution of the ion transport deficit in sweat gland reabsorptive duct. The gains in FEV_1_ over a 24-week period are 4%–8% for Class III mutations, which is very comparable to other successful therapies such as Dornase alfa (DNAse—mucolytic agent) and hypertonic saline (3%–10%). The potentiator drugs do little to sweat Cl^−^, FEV_1_, or exacerbation rate in ΔF508 mutations, which aligns with expectations, since this Class II mutation where no gene product is present in the plasma membrane, and therefore no function to potentiate. However, more recent combinatorial therapies have proven more successful.

### Extension of traditional classification system with theratyping

The traditional classification of CFTR mutations based on cellular protein handling is limited by the correlation between functional impact at the tissue level and the class of the mutation ascribed. Since vectorial ion transport and fluid secretion are dependent upon the time-dependent net flow of ions across the epithelium, it can be difficult to translate a single protein processing dysfunction up to the tissue level. Complex combinatorial molecular/cellular phenotypes are not easily categorized and mutations can display multiclass issues while responding differentially to drug therapies. Further complexity is added, since the drug therapies are also combinatorial, where multiple pharmacotherapies targeted at different molecular processes are required to restore function. For these reasons, the use of expression systems combined with a functional classification system based on pharmacological responses has been suggested.^63,64^ This is not a replacement of the original classification system but a functional expansion. Much like receptor classification based on ranked functional outcomes to a series of agonists, a somewhat analogous approach has been suggested for CFTR mutations. Since the clinical significance of any therapy depends upon the functional outcome of the putative drug therapies and not the classification of the mutant protein per se, a system of “theratyping” has been suggested. Theratyping adds to the traditional classification system by layering the measurement of the functional restoration possible using specified drug therapies (and combinations thereof), and this approach has already been successful. Indeed, the FDA’s approval of modulators in persons with several rare CFTR variants was based, in part, upon functional outcomes in response to combinations of therapeutics in cultured human cells and heterologous expression systems. The models in which these drugs can be reproducibly and reliably tested are limited, and human bronchial airway epithelial cells and transfected cell lines have been a mainstay of drug screening for CF research.^64^ These in vitro studies have been crucial during drug development, since traditional clinical trials are limited by the small populations of rare variants and the array of combinations demonstrated by compound heterozygotes in the CF population. In these circumstances, theratyping using cultured airway cells or heterologous expression systems may be the only viable approach.

## FDA approved pharmacotherapies

### Ivacaftor (Kalydeco)

Kalydeco is indicated for patients aged 2 years and older who have one mutation in the CFTR gene (Class III or IV) that is responsive to drug treatment based on clinical and/or in vitro data. In May 2017, the FDA expanded the approved use of Kalydeco, increasing the indication from the treatment of 10 mutations to 33. The expansion of the indication was approved despite the lack of further clinical data. Since collecting clinical data is difficult with Orphan diseases such as CF, a novel approach was used that relied on evidence from laboratory-based in vitro assay data where specified CFTR mutations showed gain of function at the single channel level and would likely translate to clinical benefit. Moreover, the in vitro data were also able to identify CFTR mutations that were not responsive. This approach was possible because there is a large efficacy and safety database for Kalydeco in existence. The primary efficacy endpoint for studies of Kalydeco was improvement in lung function, as determined by the mean change in FEV_1_ over a 24 week period. In all clinical studies, treatment with Kalydeco resulted in a significant improvement in FEV_1_^65,66^ (see Table 1).

Kalydeco is available as tablets or oral granules which have to be taken with fat-containing food twice a day. Common adverse effects include headache, upper respiratory tract infection, abdominal pain, diarrhea, rash, nausea, and dizziness. At higher doses, there is an increased risk of elevated transaminases and pediatric cataracts. Coadministration with strong cytochrome 3A4 inducers substantially decreases exposure of Kalydeco and is not recommended. In addition, the cost of Kalydeco is considerable (~$311,000 USD per patient per year), which leads to stringent assessment of its clinical benefit.^67,68^

### Tezacaftor plus ivacaftor (Symdeko)

Symdeko is a combination of tezacaftor and ivacaftor. Tezacaftor facilitates the cellular processing and trafficking of normal and specified mutant forms of CFTR to increase the amount of mature CFTR protein delivered to the cell surface. Ivacaftor is a CFTR potentiator that facilitates increased Cl^−^ transport by potentiating the channel-open probability of the CFTR protein at the cell surface. Ivacaftor can potentiate the CFTR protein delivered to the cell surface due to the action of tezacaftor, leading to a further enhancement of Cl^−^ transport than either agent alone.^67^

Symdeko is indicated for the treatment of CF patients aged 12 years or older who are homozygous for the ΔF508 mutation. Symdeko is packaged as two tablets. One tablet, a combination of tezacaftor and ivacaftor, is taken orally in the morning. The second tablet (ivacaftor only) is taken orally in the evening. The efficacy of Symdeko was evaluated in three clinical trials which used either improvement in ppFEV_1_ or mean absolute change in FEV_1_ as the primary endpoints (see Table 1). All trials demonstrated statistically significant improvements.^67–69^

Symdeko is well tolerated and the most commonly reported adverse effects are headache, nasopharyngitis, nausea, sinus congestion, and dizziness. More serious adverse effects include increased transaminases and an increased risk of cataracts. As with the other agents, there is a potential for drug interactions due to effects on the cytochrome enzymes.^67,69^

### Lumacaftor plus ivacaftor (Orkambi)

Orkambi is a treatment for CF that is approved for use in patients 12 years and older who are homozygous for the ΔF508 mutation. It is a combination therapy containing lumacaftor and ivacaftor. Again, this is a combined corrector/potentiator therapy. Modest efficacy has been observed using a regimen of two tablets (each containing lumacaftor and ivacaftor) taken orally every 12 h.^69^ The primary efficacy endpoint in clinical trials was a change in lung function as determined by improvement in FEV_1_. In all trials, treatment with Orkambi resulted in a statistically significant improvement (see Table 1).^67^

Orkambi is available at a lower cost than Kalydeco, which may facilitate its prescription to patients. Tolerability is high, and the common adverse effects include shortness of breath, nasopharyngitis, upper respiratory tract infections, nausea, diarrhea, vomiting, and gas. Both drugs interact with cytochrome enzymes and there is potential for drug interactions.^67,69^

Both Orkambi and Symdeko have shown only very modest effects in homozygous ΔF508 patients and have been more effective in compound heterozygous patients with Class II and Class III/IV mutations or are ΔF508 heterozygous. However, significant improvements have been made in ΔF508 homo- and heterozygous patients with a new triple therapy named Trikafta.

Trikafta is a recently approved (Fall 2019) triple therapy (elexacaftor/ivacaftor/tezacaftor) for use in patients aged >12 years, which is ΔF508 compound heterozygous or ΔF508 homozygous, and this represents a big sub-group of all CF patients (~90% CF patients in the United States). Two trials were conducted; the first using compound heterozygous ΔF508/with an alternative mutation that results in no protein or protein that is unresponsive to Symdeko or Kalydeco, and the second population was composed of CF patients homozygous for ΔF508. In these studies, an improvement of 10%–13% in predicated FEV_1_ was seen in both populations at 4 weeks and was maintained to 24 weeks in ΔF508 homozygous trial. Sweat Cl^−^ was significantly reduced with this therapy, and two important clinical indexes were also significantly improved; BMI increased by ~5% and the calculated exacerbation rate was reduced by 63%. This was an important development in CF pharmacotherapy, since these patients had been unresponsive to prior modulator regimens^1,7^ and this represents one of the most significant developments in CF pharmacotherapy and the advent of true combinatorial therapy.

## Conclusion

In conclusion, the shift in focus from symptom treatment and management to correction of the primary ion transport defect in CF is an exciting new era in the management of CF patients. Potentially, the systemic correction and potentiation of CFTR protein has huge implications for patient quality of life, where the pill burden, time in therapy, and hospital exacerbations may all be significantly reduced. The focus on pharmacotherapy, prior to the development of CF symptoms, may also significantly delay or reduce reliance on antimicrobial therapies and the associated complications of the development of drug resistance in those patients. Ultimately, with these pharmacotherapies, there is promise for a significantly increased quality of life, reduced exacerbations, and delayed emergence of symptoms in the natural history of a CF patients’ lifespan.
