# Erratum to “Epithelial vectorial ion transport in cystic fibrosis: Dysfunction, measurement, and pharmacotherapy to target the primary deficit”

**DOI:** 10.1177/2050312120974970

**Published:** 2020-12-15

Clunes LA, McMillan-Castanares N, Mehta N, et al. Epithelial vectorial ion transport in cystic fibrosis: Dysfunction, measurement, and pharmacotherapy to target the primary deficit, *SAGE Open Medicine*, 2020; 8: 1–13. DOI: 10.1177/2050312120933807.

The above referenced article, when was originally published, Figure 1 appeared as Figure 2 and figure 2 was missing from the article.

The online version of this article has now been updated.

